# Salience Network Connectivity in Anorexia Nervosa with and without Body Image Distortion

**DOI:** 10.1192/j.eurpsy.2026.10725

**Published:** 2026-07-31

**Authors:** P. Holštajn Zemánková, S. Yennampelli

**Affiliations:** 1 Department of Psychiatry, Masaryk University, Brno, Czech Republic; 2 Cambridge University, Cambridge, United Kingdom

## Abstract

**Introduction:**

Anorexia nervosa (AN) is a severe psychiatric condition in which body image distortion is a particularly prominent symptom. Such distortions can reduce motivation to engage in treatment and hinder weight recovery. Importantly, the presence and intensity of body image distortion vary across individuals with AN, suggesting heterogeneity in the disorder’s neurobiological underpinnings. The salience network, which detects and prioritizes emotionally and physiologically relevant stimuli and processes internal body signals as well as external visual information, may contribute to body image distortions.

**Objectives:**

This study aimed to examine resting-state functional connectivity (rsFC) of the anterior cingulate cortex (ACC), a hub of the salience network involved in self-monitoring and cognitive control, in hospitalized female AN patients with (BD+) and without (BD−) pronounced body image distortion, to clarify its neurobiological mechanisms.

**Methods:**

Twenty-six women diagnosed with restrictive-type AN underwent resting-state functional MRI scanning. Based on their scores from the Stunkard Figure Rating Scale, they were divided into two subgroups: BD+ (n=12) and BD− (n=14). Functional imaging data were preprocessed and analyzed using the CONN toolbox, employing a salience network connectivity analysis with the ACC as the seed region.

**Results:**

In cross-sectional analyses controlling for age, BD+ AN patients exhibited altered resting-state connectivity of the ACC compared to BD− AN patients. Specifically, BD+ patients showed significantly reduced rsFC between the ACC and the right superior and inferior lateral occipital cortex (sLOC, iLOC), as well as the bilateral superior/middle frontal gyrus (SFG/MFG). Conversely, they demonstrated significantly increased rsFC between the ACC and the right cerebellum (Crus I/II) (all p < 0.05, FDR-corrected).

**Conclusions:**

The findings indicate that impaired top-down cognitive control over visual body perception and self-referential processing, coupled with over-engagement of cerebellar self-monitoring networks, may contribute to persistent body image distortions in AN. These findings align with the Allocentric Lock Hypothesis, suggesting that BD+ patients rely on internalized, memory-based representations rather than updating perceptions based on current visual feedback. The results highlight potential targets for personalized neuromodulation interventions in AN treatment.

**Disclosure of Interest:**

None Declared

